# Quality of life and associated factors among patients with breast cancer under chemotherapy at Tikur Anbessa specialized hospital, Addis Ababa, Ethiopia

**DOI:** 10.1371/journal.pone.0222629

**Published:** 2019-09-20

**Authors:** Anissa Mohammed Hassen, Girma Taye, Muluken Gizaw, Foziya Mohammed Hussien

**Affiliations:** 1 Department of Public Health, College of Medicine and Health Science, Wollo University, Dessie, Ethiopia; 2 School of Public Health, College of Medicine and Health Science, Addis Ababa University, Addis Ababa, Ethiopia; University of South Alabama Mitchell Cancer Institute, UNITED STATES

## Abstract

**Background:**

Breast cancer is the most common cancer affecting women in Ethiopia with increasing burden, and chemotherapy treatment produces a detrimental effect on individual wellbeing. Since last few years quality of life has been the primary goal of cancer treatment, yet little research has been conducted on quality of life of breast cancer patients under chemotherapy.

**Objective:**

To determine the quality of life and associated factors among patients with breast cancer under chemotherapy at Tikur Anbessa specialized hospital, Addis Ababa, Ethiopia.

**Methods:**

Institution based cross-sectional study was conducted on 404 patients with breast cancer, who took at least one cycle of chemotherapy treatment using face to face interview at oncology unit of Tikur Anbessa specialized hospital day care center from February to April 2018. The validated Amharic version of European organization for research and treatment of cancer core 30 (EORTC QLQ-C30) and quality of life questionnaire specific to breast (QLQ-BR23) was used to measure health related quality of life. Both descriptive and inferential statistics were used. For the purpose of interpretation quality of life score was dichotomized in to two using the calculated mean score, which is 53 as a cutoff point, then, bi-variable and multivariable logistic regression was used to describe association between dependent and independent variables. Hence, patients who score above 53 for quality of life were considered to have good quality of life.

**Result:**

Of the total sample, overall response rate was 99.77%. The average quality of life score of patients with breast cancer under chemotherapy treatment was 52.98 (SD = 25.61). Majority of patients had scored poor in emotional functioning, sexual functioning, and financial difficulties. Educational status of college and above, being divorced, higher household income, higher scores of physical and social functioning were associated with significantly improved (better) quality of life. Lower scores of fatigue, insomnia, financial difficulties and systemic therapy side effects all were associated with better scores of quality of life of breast cancer patients. Whereas, patients receiving < = 2 cycles of chemotherapy had significantly lower scores of quality of life.

**Conclusion and recommendation:**

Quality of life of breast cancer patients under chemotherapy treatment is poor in comparison with the reference data and international findings. Therefore, quality of life assessment should be incorporated in patient’s treatment protocol. And financial aids may significantly improve the quality of life of breast cancer patients under chemotherapy treatment.

## Introduction

Worldwide breast cancer is a major life threatening and the major public health problem of great concern. It is estimated that 1.7 million new cancer cases were diagnosed worldwide in 2012. This makes it the second most common form of cancer following lung cancer [1, 2].

According to world health organization (WHO) 2015, annually around 60,000 new breast cancer cases are diagnosed in Ethiopia, and the major obstacles in the country are lack of trained health professionals and oncologists[3]. A study conducted to assess the pattern of cancer in Tikur Anbessa Specialized Hospital Oncology Centre in Ethiopia from 1998–2010 showed that, breast cancer has been the second most common form of cancer following cervical cancer accounting 26% [4].

To increase the survival rates and reduce the risk of recurrence breast cancer patients face different types of treatment for the disease, such as surgeries and radiotherapy and chemotherapy treatments, frequently associated to adverse side effects [5]. The majority of breast cancer patients in Ethiopia (83%) received chemotherapy treatment as a front-line therapy, as an adjuvant to surgery or radiotherapy and even in palliative care [6].

During the past four decades, Quality of life (QOL) has become an important outcome in medical and psychological research. Increasingly there has been a growing recognition that maintaining or improving the quality of life for cancer patients is an important treatment goal, since, it is well described previously that, clinical data only show small correlations with patients’ judgments [7, 8].

Being diagnosed with breast cancer is a very stressful event and has tremendous consequences for most persons who experience it, affecting all aspects of life and the temporary side effects associated with the treatment may influence the patients’ health related quality of life during treatment. In the case of breast cancer, the initial treatment usually consists of surgery, and after the operation many patients are recommended one or more additional treatments including radiotherapy, chemotherapy, and hormonal treatment. All these factors may, of course, impact the patients’ quality of life thus compromising the quality of life [9–11]. Moreover; the incurable nature of breast cancer along with its reoccurrence causes psychological distress to clients than the diagnosis of primary breast cancer that in turn affects the quality of life of these patients [12, 13].

As described in detail in the previous study [7], Assessing quality of life of patients has numerous benefits including the ability to provide clinicians and patients with accurate expectations about the likely impact of treatments on wellbeing and functioning, the ability to identify common problems that will need to be addressed, and the ability to identify therapies and interventions effective in addressing these problems. In addition, findings suggest that QOL data may improve clinicians’ ability to predict treatment response and survival time in certain contexts. Besides, numerous studies have found that a better quality of life measure is associated with longer survival of patients in different types of cancer [14, 15].

In Ethiopia, little research has been conducted to evaluate quality of life of breast cancer patients[16]. Considering the increasing prevalence of breast cancer and its destructive effect on QOL and low local reports pertaining to QOL of breast cancer patients’ under chemotherapy treatment, this study aims to evaluate the quality of life and associated factors among breast cancer patients’ under chemotherapy treatment using the validated questionnaire.

## Methods and materials

### Study design, participants and setting

A facility-based, cross-sectional study design was employed from February to May 2018, at adult oncology unit of Tikur Anbessa specialized hospital (TASH) day care Centre, located in Addis Ababa, Ethiopia. The study population were all female breast cancer patients who were under chemotherapy at outpatient department of TASH oncology unit. Female breast cancer patients greater than 18 years old who took at least one cycle of chemotherapy were included. And patients who were severely ill and unable to communicate during the data collection were excluded from the study. All patients with breast cancer who visited the day care center of TASH for chemotherapy treatment during the data collection period were included consequently. Thus, a total of 404 breast cancer patients who visited the day care Centre of TASH for chemotherapy treatment were taken consequently.

### Data collection tools and procedures

The validated Amharic version of European organization for research and treatment of cancer quality of life questionnaire core 30 (EORTC QLQ C-30) was used to measure breast cancer patients’ health related quality of life in addition to the socio demographic and clinical characteristics questionnaire. Besides, European organization for research and treatment of cancer quality of life questionnaire specific for breast cancer (EORTC QLQ BR23) was used to assess specific factors of breast cancer patients’ QOL. The EORTC QLQ C-30 questionnaire is a multi-item questionnaire aimed to address the health related QOL of cancer patients in general. It has 30 questions, composed of five multi item functional subscales: physical, role, emotional, social and cognitive functioning; three multi item symptom scales measuring fatigue, pain, and emesis; a global health status subscale (quality of life); and six single items to assess financial impact and symptoms such as dyspnoea, sleep disturbance, appetite, diarrhoea, and constipation. Likewise, the QLQ- BR23, which assesses the QOL of breast cancer patients, has 23 items assessing disease symptoms, side effects of treatment, body image, sexual functioning and future perspective [17]. The data was collected through face to face interview and variables on clinical characteristics were extracted from medical charts at the oncology unit. The global health status (quality of life) was the dependent variable, while, socio-demographic variables, like age, educational status, marital status, occupation, and household income: clinical variables like stage at diagnosis, time since diagnosis, number of chemotherapy session taken, previous treatment taken and the tumour size are the independent variables. To maintain the quality of data, training was given to the data collectors for two days on how to fill the questionnaire and clarification of the whole study tools, variables and research ethics. Continuous monitoring and supervision was done by the principal investigator for completeness of the data. Moreover, pre-test was done on 20 patients to identify clarity and applicability of the tools, and to provide feedback about the questionnaire.

### Statistical analysis

Data was entered cleaned and coded into Epi-data 4.2 Software and then exported to Statistical Package for the Social Science (SPSS Version 20.0) for analysis. Simple descriptive statistics such as frequencies, mean, and standard deviation (SD) was calculated. All of the scales and single-item measures range in score from 0 to 100. A high scale score represents a higher response level. Thus, a high score for a functional scale represents a high/ healthy level of functioning; a high score for the global health status / QOL represents a high QOL, but a high score for a symptom scale / item represents a high level of symptomatology / problems [18]. The raw scores were transformed to scores ranging from 0 to 100 by using the following formula.

Rawscore=RS=(I1+I2+⋯+Inn)

Apply the linear transformation to 0–100 to obtain the score S,

$$\text{Functional}\;\text{scale}\;:\;\text{S}=\left(1-\frac{RS-1}{range}\right)\times 100$$

$$\text{Symptom}\;\text{scale}\;:\;\text{S}=\left(\frac{RS-1}{range}\right)\times 100$$

$$\text{Global}\;\text{health}\;\text{status}\;/\text{QOL}\;:\;\text{S}=\left(\frac{RS-1}{range}\right)\times 100$$

After transformation of the raw score, based on the calculated mean score of the study participants’ quality of life score, it has been dichotomized in to two using 53 as a cutoff point. Therefore; using 53 as a cutoff point, it was dichotomized in to “poor QOL” and “good QOL” in which a score below 53 for functional and global health status/QOL and a score above 53 for symptom scale indicates poor QOL. After dichotomization of the transformed score, bi-variable and multivariable logistic regression was used to assess the association of QOL with socio demographic and clinical variables and functional and symptom scales of EORTC QLQ C-30 and QLQ BR 23. As a result, crude and adjusted odds ratio with 95% confidence interval was calculated. A p-value of less than or equal to 0.05 was considered significant.

Ethical clearance and approval letter was obtained from the ethical clearance committee of Addis Ababa University (AAU) College of health science, school of public health ethical review committee to conduct the research. An official letter of approval was written to TASH oncology unit. Informed written consent was obtained from the study participants after clearly introducing the purpose, the benefits and risks of the study. Moreover, the participants assured that no harm occur to them by not participating in the study. Confidentiality was secured by avoiding writing the identification of the participant’s name.

## Results

### Socio demographic and clinical characteristics of study participants

There were 404 eligible respondents during the study period. Of these, only 1 (0.25%) participant refused to participate and was excluded from the study giving a response rate of 99.75%.

The mean age of the study participants were (Mean ± SD) 44 ± 11.78. Majority of participants were unable to read and write (25.8%), housewives (61.5%), married (56.8%) and residents outside of Addis Ababa. There were 351 patients who had previous exposure to different cancer treatments. Among them, 79.3% had undergone breast surgery. Concerning the current exposure to chemotherapy treatment, 188 (46.7%) participants were in their first three cycle of chemotherapy treatment. Most of the patients (45.4%) were diagnosed with stage IV cancer, whereas only 4% with stage I cancer. Approximately 10.9% of patients had recurrent breast cancer (breast cancer that comes back after initial treatment). More than half of participants seek treatment within 12 months of diagnosis. (Table 1).

**Table 1:** Socio demographic and clinical characteristics of breast cancer patients under chemotherapy, Addis Ababa, Ethiopia 2018.

Variables	Category	Number of participants(n)	Percent (%)
Age	<35	123	30.5
	35–50	177	43.9
	51–65	85	21.1
	66–80	18	4.5
Educational status	Unable to read and write	97	24.1
	Able to read and write	39	9.7
	Primary education	87	21.6
	Secondary education	104	2.8
	College and above	76	18.9
Occupation	Housewife	248	61.5
	Government employee	60	14.9
	Non-government employee	13	3.2
	Farmer	28	6.9
	Merchant	34	8.4
	Other	20	5
Marital status	Married	229	56.8
	Single	40	9.9
	Divorced	56	13.9
	Husband died	78	19.4
Residence	Addis Ababa	182	45.2
	Out of Addis Ababa	221	54.8
Monthly income	< = 800	102	25.3
	801–1800	104	25.8
	1801–4000	98	24.3
	>4000	99	24.6
Stage at diagnosis	Stage I	16	4
	Stage II A	53	13.2
	Stage IIB	92	22.8
	Stage IIIA (T3,N1,M0)	23	5.7
	Stage III *	160	39.7
	Stage IV	59	14.6
Tumor size	Tx (Not assessed)	12	3
	T1 (< 2 cm)	47	11.7
	T2 (2 cm-5 cm)	145	36
	T3 (> 5 cm)	94	23.3
	T4 (Any size with extension to chest wall)	105	26.1
Comorbid disease	Hypertension	50	65.78
	Diabetes mellitus	24	31.57
	HIV AIDS	8	10.52
	Asthma	12	15.78
	Cardiac disease	4	5.26
Previous exposure to breast cancer treatment	Surgery alone	261	79.3
	Surgery and radiotherapy	56	17.0
	Surgery and chemotherapy	13	4.0
	Surgery, radiotherapy and chemotherapy	21	6.4
Sequence of chemotherapy cycle	2^nd^ cycle	100	24.8
	3^rd^ cycle	88	21.8
	4^th^ cycle	50	12.4
	5^th^ cycle	58	14.4
	6^th^ cycle	44	10.9
	7^th^ cycle	38	9.4
	8^th^ cycle	25	6.2

*stage III other than T3, N1, M0

The mean global health status (quality of life) of the study participants was 52.98 with standard deviation of 25.61. Two hundred nineteen (54.3%) participants had scored less than 53 and had poor global health status/ quality of life; while the rest had scored greater than or equal to 53 hence had good quality of life.

In the EORTC QLQ C-30, The functional scale of study participants ranged from a mean of (± SD) 47.61 ± 25.83 for emotional functioning to a mean of 80.06 ± 22.89 for cognitive functioning. Majority of participants had poor emotional (71.5%) and social functioning (59.3%). Whereas, only 17.4% of partipants had poor cognitive functioning. Concerning the symptom scale; 79.2% of participants had faced financial difficulties and more than half of participants (57.3% and 53.6) suffered from fatigue and constipation respectively. On the contrary, nausea and vomiting was the least affected symptom scale with majority of participants 266 (66%) didn’t experience this symptom.

The EORTC QLQ B-23 functional scale ranged from a mean (SD) of 55 (38.48%) for future perspective to a mean of 89 (21.10) for sexual functioning. The most affected functional scale was sexual functioning in which 85.8% had poor sexual functioning; whereas, body image was the least affected in which only 16.6% participants had poor body image. On the other hand, the most unbearable symptom was breast symptom in which 663 (90.1%) participants had suffered with breast symptoms.

The association between socio-demographic and clinical variables with QOL is shown in Table 2. Educational status, marital status, income and sequence of chemotherapy cycle had significant association with health related QOL. Breast cancer patients with educational status of college and above had 1.6 times good QOL than patients with no formal education (OR = 1.6, P<0.041). Besides, Divorced mothers were more likely to have good QOL than singles (OR = 1.6, P<0.021), similarly, in comparison with those breast cancer patients who earned < = 800 ETB, those breast cancer patients who had an income between 1801–4000 and greater than 4000 had 3 fold times good QOL (OR = 3.8, p <0.002) (OR = 7.9, p<0.0001) respectively. Quality of life also significantly got better with chemotherapy cycle. Breast cancer patients who took more than 3 cycles of chemotherapy treatment had 2.4 times better QOL than those who took less than or equal to 3 cycles of chemotherapy treatment (OR = 2.4, p<0.005).

**Table 2:** Binary and multivariable logistic regression of socio-demographic and clinical variables with global health status (QOL) of breast cancer patients under chemotherapy at TASH Addis Ababa, Ethiopia, 2018.

Variable category	Poor QOL N (%)	Good QOL N (%)	COR (95%CI)	AOR (95% CI)	P value
**Age**					
<35	63 (28.8)	60 (32.6)	1	1	
36–50	92 (42.0)	85 (46.2)	0.9 (0.61–1.53)	1.4 (0.76–2.42)	0.310
51–65	49 (22.4)	36 (19.6)	0.8(0.44–1.35)	1.5 (0.67–3.18)	0.304
>66	15 (6.8)	3 (1.6)	0.2 (0.06–0.76)	0.5 (0.11–2.42)	0.401
**Educational status**					
Unable to read & write	65 (29.7)	32 (17.4)	1	1	
able to read &write	21 (9.6)	18 (9.8)	1.7 (0.81–3.72)	1.5 (0.55–3.89)	0.446
Primary education	51 (23.3)	36 (19.6)	1.4 (0.78–2.62)	0.8 (0.36–1.64)	0.497
Secondary education	51 (23.3)	53 (28.8)	2.1 (1.19–3.74)	0.96 (0.44–2.09)	0.927
College and above	31 (14.2)	45 (24.4)	2.9 (1.58–5.49)	1.6 (1.01–3.03)	0.041*
**Marital status**					
Single	24 (11.0)	16 (8.7)	1	1	
Married	112 (51.1)	117 (63.6)	1.6 (1.12–3.10)	1.6 (0.68–3.70)	0.277
Divorced	31 (14.2)	25 (13.6)	1.2 (0.53–2.75)	1.6 (1.12–5.54)	0.021*
Husband died	52 (23.7)	26 (14.1)	0.7 (0.34–1.65)	1.5 (0.52–4.30)	0.463
**Income**					
< = 800	84 (38.4)	18 (9.8)	1	1	
801–1800	67 (30.6)	37 (20.1)	2.6 (1.35–4.93)	1.9 (0.89–4.09)	0.009
1801–4000	45 (20.5)	53 (28.8)	5.5 (2.88–10.48)	3.8 (1.63–8.91)	0.002*
>4000	23 (10.5)	76 (41.3)	15.4 (7.74–30.7)	7.9 (3.13–20.02)	0.0001*
**Chemotherapy sequence**					
< = 3 cycles	142 (64.8)	46 (25.0)	1		
>3 cycles	77 (35.2)	138 (75.0)	5.5 (3.58–8.54)	2.4 (1.29–4.41)	0.005*
**Stage of the disease**					
Early stage	32 (18.1)	37 (22.2)	1	/	
Late stage	145 (81.9)	130 (77.8)	0.8 (0.46–1.34)	/	
**Time since diagnosis**					
<12 months	138 (63.0)	111 (60.3)	1	/	
13–24 months	12 (5.5)	19 (10.3)	1.9 (0.91–4.22)	/	
25–36 months	11 (5.0)	9 (4.9)	1.0 (0.41–2.54)	/	
37–48 months	7 (3.2)	8 (4.3)	1.4 (0.50–4.04)	/	
>48 months	51 (23.3)	37 (20.1)	0.9 (0.55–1.47)	/	

**/** indicates: not included in the model

* indicates significant association

Tables 3 and 4 shows the multivariable regression analysis for the association of functional and symptom scales of EORTC QLQ C-30 and QLQ BR23 with global health status (QOL). Breast cancer patients who had good physical functioning had 1.6 times good QOL than those patients who had poor physical functioning (OR = 1.6, p<0.001), similarly patients who had good social functioning had about 50% more times good QOL than patients whose social functioning was poor (OR = 1.5, p<0.024).

**Table 3:** Bivariate and multivariable logistic regression of EORTC QLQ C-30 functional and symptom scales with global health status (QOL) of breast cancer patients under chemotherapy at TASH Addis Ababa Ethiopia, 2018.

Variables		Poor QOL n (%)	Good QOL n (%)	COR (95% CI)	AOR (95% CI)	P value
**Functional scale**						
**Physical functioning**	Poor	77 (35.2)	36 (19.6)	1	1	
	Good	142 (64.8)	148 (80.4)	2.2 (1.41–3.52)	1.6 (1.08–5.06)	0.001*
**Role functioning**	Poor	115 (52.5)	62 (33.7)	1	1	
	Good	104 (47.5)	122 (66.3)	2.2 (1.45–3.26)	1.3 (0.69–2.26)	0.422
**Emotional functioning**	Poor	73 (33.3)	42 (22.8)	1	1	
	Good	146 (66.7)	142 (77.2)	1.7 (1.08–2.64)	0.9 (0.54–1.78)	0.956
**Cognitive functioning**	Poor	43 (19.6)	27 (14.7)	1	/	
	Good	176 (80.4)	157 (85.3)	1.4 (0.84–2.41)	/	
**Social functioning**	Poor	141 (64.4)	98 (53.3)	1	1	
	Good	78 (35.6)	86 (46.7)	1.6 (1.06–2.37)	1.5 (1.09–45)	0.024*
Symptom scale						
**Fatigue**	Poor	146 (66.7)	85 (46.2)	1	1	
	Good	73 (33.3)	99 (53.8)	2.3 (1.56–3.49)	1.9 (1.17–5.09)	0.007*
**Nausea &vomiting**	Poor	88 (40.2)	49 (26.6)	1	1	1
	Good	131 (59.8)	135 (73.4)	1.9 (1.21–2.83)	0.5 (0.14–1.91)	0.143
**Pain**	Poor	96 (43.8)	48 (26.1)	1	1	1
	Good	123 (56.2)	136 (73.9)	2.2 (1.45–3.38)	1.4 (0.34–5.52)	0.653
**Dyspnea**	Poor	108 (49.3)	57 (31.0)	1	/	
	Good	111 (50.7)	127 (69.0)	2.2 (0.43–3.26)	/	
**Insomnia**	Poor	111 (50.7)	54 (29.3)	1	1	
	Good	108 (49.3)	130 (70.7)	2.5 (1.63–3.74)	8.3 (1.06–15.1)	0.61
**Appetite loss**	Poor	127 (58.0)	84 (45.7)	1	/	
	Good	92 (42.0)	100 (54.3)	1.6 (0.10–2.44)	/	
**Constipation**	Poor	131 (59.8)	85 (46.2)	1	1	
	Good	88 (40.2)	99 (53.8)	1.7 (1.17–2.58)	4.7 (0.65–34.4)	0.126
**Financial difficulties**	Poor	184 (84.0)	135 (73.4)	1	1	
	Good	35 (16.0)	49 (26.6)	1.9 (1.17–3.11)	3.5 (1.63–7.57)	0.001*

*significant at P<0.05

**Table 4:** Bivariate and multivariable logistic regression of EORTC QLQ BR23 functional and symptom scales with global health status (QOL) of female breast cancer patients under chemotherapy at TASH Addis Ababa Ethiopia, 2018.

Variables		Poor QOL N (%)	Good QOL N (%)	COR (95% CI)	AOR (95% CI)	P value
**Functional scale**						
Body image	Poor	30 (13.7)	37 (20.1)	1	/	
	Good	189 (86.3)	147 (79.9)	0.6 (0.37–1.06)	/	
Sexual functioning	Poor	194 (88.6)	156 (84.8)	1	1	
	Good	25 (11.4)	28 (15.2)	1.4 (1.04–2.48)	0.7 (0.36–1.65)	0.504
Sexual enjoyment	Poor	4 (11.1)	10 (25.6)	1	/	
	Good	32 (88.9)	29 (74.4)	0.4 (0.10–1.28)	/	
Future perspective	Poor	122 (55.7)	83 (45.1)	1	1	
	Good	97 (44.3)	101 (54.9)	1.5 (1.03–2.27)	1.6 (0.95–2.64)	0.077
**Symptom scale**						
Systemic therapy side effects	Poor	139 (63.5)	132 (71.7)	1	1	
	Good	80 (36.5)	52 (28.3)	0.68 (0.45–0.98)	1.5 (1.03–7.56)	0.048*
Breast symptoms	Poor	193 (88.1)	170 (92.4)	1	/	
	Good	26 (11.9)	14 (7.6)	0.6 (0.31–1.21)	/	
Arm symptoms	Poor	47 (21.5)	21 (11.4)	1	1	
	Good	172 (78.5)	163 (88.6)	2.1 (1.22–3.70)	0.9 (0.46–1.96)	0.906
Upset by hair loss	Poor	54 (27.8)	38 (23.0)	1	/	
	Good	140 (72.2)	127 (77.0)	1.2 (0.79–2.08)	/	

/ indicates: not included in the model

*significant at p<0.05

Regarding the symptom scale, those who were classified as having less symptoms of fatigue had 90% more likely to have good QOL than those who were classified as poor fatigue symptom (OR = 1.9, P<0.017). Likewise; compared with those patients who had high financial difficulties, those patients who had less financial difficulty (good financial status) had more than 3 fold times more likely to have good QOL (OR = 3.5, p<0.001).

Those breast cancer patients who did not experience poor systemic therapy side effects had 1.5 times good QOL than breast cancer patients who experienced poor systemic therapy side effects. Though not statistically significant, breast cancer patients who had good future perspective had 60% times good QOL than those who had poor future perspective.

## Discussion

The mean QOL score of the study participants obtained in this study was consistent with previous studies done in Ethiopia, Morocco, Nigeria, and Nepalese breast cancer patients [16, 19–21]. However, it was lower than the EORTC QLQ reference value manual for breast cancer patients (61.8 ± 24.6) indicating poor QOL [22]. The reference value manual is based on pretreatment QOL data only. Therefore, the reason for poor QOL might be due to the different treatment side effects that most patients had been taking including surgery, radiotherapy, and chemotherapy. The mean score of QOL of breast cancer patients was also lower than studies done in Iran, Sweden, Bahrain, India, Australia, Brazil, and Kenya [12, 23–28]. The discrepancy for this result might be, due to the difference in socio-demographic characteristics of study participants and different study designs employed. Unlike some studies mentioned here, this study did not compare the QOL of breast cancer patients at different time intervals, but assessed at a point in time. Besides, patients’ recruitment method can explain the difference, in which some other studies enrolled breast cancer patients undergoing different forms of treatment, but this study only assessed patients under chemotherapy.

Moreover, most of the patients in this study are at stage III and above which might put the patients on frequent visit to the hospital which in turn lead to poor QOL. Besides, majority of the patients in this study come from outside of Addis Ababa traveling long distance to the hospital and wait for longer periods of time to get appropriate treatment due to long queue of patients waiting for treatment at the hospital. This in turn may cause psychological and economic stress leading to poor QOL.

The mean score for physical, role and social functioning was lower than the EORTC QLQ reference value manual for breast cancer but, similar in other functional scales in the EORTC QLQ C-30 questionnaire [22]. Emotional functioning was the most affected functional scale; While Physical and role functioning was the least affected functional scales. This is similar with other studies [23–27]. The reduced emotional functioning might be due to the role of women in Ethiopia is to take care of the family, so when they get sick, they perceive disruption in their usual role and worry more about their family. In addition, they have much concern for their children’s future, resulting in poor emotional functioning.

Despite government subsidization program for those patients who are unable to pay for their chemotherapy treatment expenses, patients scored poor in financial difficulties. This is contrary to other studies in Sweden, Brazil, and Iran [23, 24, 26]. Majority of the Ethiopian population belonged to lower‑middle class families and had one earning member who solely responsible for all family expenditure. Thus in addition to the usual household expenses, the expenses for chemotherapy treatment may pose additional financial burden. In the same category, patients scored better in nausea and vomiting. This might be because symptoms of nausea and vomiting are experienced within a week period of taking chemotherapy treatment. However, the patients have been asked about the symptoms they have experienced in the last week period. As a result, when they come for the next visit the symptoms might already been alleviated.

Regarding the EORTC QLQ BR23 scores, patients scored worse in sexual functioning. This is in consistent with previous studies [12, 20, 24, 25]. The reason for lower sexual functioning might be due to the effect of the disease (being late stage) and its treatments on psychological and physical aspects of sexuality. The breast is an organ of sexuality and fertility and loss of one breast may be loss of all these. In addition, patients wrongly believe that, having sexual intercourse during chemotherapy treatment might worsen the disease progression. As a result, they do not engage in the sexual activities leading to poor sexual functioning. Moreover, as this topic is sensitive, patients may perceive it improper to express their sexual desire. In the same category, future perspective was better than studies conducted in Brazil and Sweden [24, 26]. This might be because; Ethiopian societies may get strong social support from the families, friends, relatives, and neighbors. And it is believed that, social support has a potential to play a protective role by buffering the impact of life stress on patients and enhance QOL of breast cancer patients [20]. In addition, patients have a strong religious belief, and have a great hope of cure if they get the treatment properly. As a result, they do have less worry for their future.

It is found that, college education is associated with better QOL. This is consistent with previous studies [20, 23, 28, 29]. This might be because educated persons might get better opportunity for different salaried employment positions, more access to economic resource, interaction with other people and sense of self control.

The present study shows that high household income is associated with better QOL. Higher socioeconomic status has been linked to many aspects of better care of patients such as having less worry about the financial difficulties and being absent from work [30]. Similarly, it is well established that patients who experience economic hardship are at risk for developing distress. This study has found that patients who have financial difficulties have poor QOL. This result is in line with previous studies in Iran, Pakistan, Nigeria, and Ethiopia [16, 19, 23, 31]. The finding is not surprising, because chemotherapy is a prolonged and expensive treatment which creates financial burden among the breast cancer patients. Besides, in countries like Ethiopia, this is even worse as there is only limited number of facility available for chemotherapy treatment causing all the patients to travel long distance to get the treatment adding to the already elevated financial burden on the patients.

Similar to studies in Iran, Pakistan, Nigeria, and Ethiopia [16, 19, 23, 31], patients with symptoms of high fatigue and sleep disturbance have poor QOL. Fatigue has been well reported to have significant impact on patient’s QOL. It has been said that many patients regard the treatment of fatigue as more important than the treatment of pain in contrast to the opinion of many physicians [32, 33]. Similarly, insomnia is associated with a number of adverse medical, social, and psychological consequences leading to QOL impairment [34].

## Conclusion

The overall quality of life of breast cancer patients under chemotherapy treatment is above average but is lower in comparison with the reference data and international findings. Moreover, participants had been affected by emotional, social and financial difficulties. Educational status, income, financial difficulties, fatigue and insomnia were significant factors that affect he QOL of breast cancer patients under chemotherapy. Therefore; the study recommends that quality of life assessment should be incorporated in patient’s treatment protocol; Emphasis should be given to empowering women through education, as it is a key tool for avoiding unemployment and tackling the psychological impact of breast cancer and financial aids may significantly improve the health of breast cancer patients. Moreover further studies are recommended to identify important determinant factors using stronger study designs.

## Supporting information

S1 File Questionnaire. (DOCX)

## References

[1] World Health Organization: Latest world cancer statistics Geneva: International Agency For Research on Cancer (IARC); 2013 [cited 2017 Oct 23]. https://www.uicc.org/iarc-release-latest-world-cancer-statistics

[2] International Agency For Resarch on Cancer; Estimated Breast Cancer Incidence, Mortality and Prevalence Worldwide in 2012: Globocan; 2012 http://globocaniarcfr/old/FactSheets/Cancers/breast-newasp.

[3] World Health Organization: Cancer—a Growing Public Health Concern for Ethiopia 2015 [cited 2017 Dec]. http://www.afrowhoint/news/cancer-growing-public-health-concern-ethiopia.

[4] Tigeneh W, Molla A, Abreha A and Assefa M. Pattern of Cancer in Tikur Anbessa Specialized Hospital Oncology Center in Ethiopia from 1998 to 2010 Int J Cancer Res Mol Mech 2015;1.1:5.

[5] Sabate E. Adherence to long term therapies: Evidence for action. World health organization (WHO). 2003:110.

[6] Kantelhardt E, Zerche P, Assefa M, Trocchi P., Addissie A, Aynalem A, et al Breast cancer survival in Ethiopia: A cohort study of 1,070 women. Int J Cancer. 2014;135:8. 10.1002/ijc.28691 24375396

[7] Paul B, Jacobsen K, and Cella D. Assessing Quality of Life in Research and Clinical Practice Cancer Network. 2002. 12380963

[8] Osoba D. Health-related quality of life and cancer clinical trials. Ther Adv Med Oncol. 2011;3(2):15. 10.1177/1758834010395342 PMC3126042 21789156

[9] Montazeri A, Vahdininia M, Harirchi I, Ebrahimi M, Khaleghi F and Jarvandi S. Quality of life in patients with breast cancer before and after diagnosis: an eighteen months follow-up study. BMC. 2008;8(330):6. 10.1186/1471-2407-8-330 PMC2588619 19014435

[10] Eisenbraun J, Scheer R, Kröz M, Schad F, and Huber R. Quality of life in breast cancer patients during chemotherapy and concurrent therapy with a mistletoe extract. Phytomedicine. 2010;18(2011):7. 10.1016/j.phymed.2010.06.013 20724129

[11] Davies N. Measuring health-related quality of life in cancer patients. Nursing Standard. 2009;23(30):8. 10.7748/ns2009.04.23.30.42.c6930 19408494

[12] Grabsch B, David M, Love A, Dean P, Raymond D, Bloch S, et al, Psychological morbidity and quality of life in women with advanced breast cancer: A cross-sectional survey. palliative and supportive care therapy. 2006;4:10. 10.1017/s1478951506060068 16889323

[13] Perry S, Kualski T, and Chang C. Quality of life assessment in women with breast cancer: benefits, acceptability and utilization. BMC. 2007;5(24):14. 10.1186/1477-7525-5-24 PMC1877797 17474993

[14] Ediebah D, Coins C, Zikos E, Quinten C, Ringash J, King M, et al Does change in health-related quality of life score predict survival? Analysis of EORTC 08975 lung cancer trial British Journal of Cancer 2014;110:7. 10.1038/bjc.2014.208 PMC4021536 24743709

[15] Sharma A, Walker L, and Monson J. Baseline Quality of Life Factors Predict Long Term Survival after Elective Resection for Colorectal Cancer. International Journal of Surgical Oncology. 2013:6. 10.1155/2013/269510 PMC3863491 24369498

[16] Bekele M. Assessing The Quality Of Life Among Patients With Breast Cancer At Tikur Anbassa Specialized Hospital, Addis Ababa, Ethiopia: Oslo and Akershus University; 2016.

[17] Aaronson N, Ahmedzai S, Bergman B, Bullinger M, Cull A, Duez N, et al The European Organisation for Research and Treatment of Cancer QLQ-C30: A quality-of-life instrument for use in international clinical trials in oncology. Journal of the National Cancer Institute 1993;85:78. 10.1093/jnci/85.5.365 8433390

[18] Fayers P, Aaronson N, Bjordal K, Groenvold M, Curran D, and Bottomley A, on behalf of the EORTC Quality of Life Group. The EORTC QLQ-C30 Scoring Manual (3rd Edition). Brussels: European Organisation for Research and Treatment of Cancer, Brussels 2011.

[19] Jaiyesimi A, Sofela E, and Rufai A. Health related quality of life and its determinants in Nigerian breast cancer patients. African Journal Of Medicine and Medical Sciences. 2007;36:8. 18390066

[20] Manandhar S, Shrestha D, Taechaboonsermsk P, Siri S, and Suparp J. Quality of Life among Breast Cancer Patients Undergoing Treatment in National Cancer Centers in Nepal. Asian Pac J Cancer Prev. 2014;15(22):5. 10.7314/apjcp.2014.15.22.9753 25520099

[21] Haddou B, Rahou K, Hanchi Z, Ouasmani F, Benazzouz B, Ahid S, et al Mesfioui Quality of Life among Moroccan Women Undergoing Treatment of Breast Cancer BJMMR. 2017;21(8):11.

[22] Scott N, Fayers P, Aaronson N, Bottomley A, Graeff A, Grønvold M, et al EORTC QLQ-C30 Reference Values 2008:427.

[23] Safaee A, Dehkordi M, Zeighami B, Tabatabaee H, and Pourhoseingholi M. Predictors of quality of life in breast cancer patients under chemotherapy. Indian Journal of Cancer 2008;45(3). 10.4103/0019-509x.44066 19018114

[24] Høyer M, Johansson B, Nordin K, Bergkvist L, Ahlgren J, Lidin-Lindqvist A, et al Health-related quality of life among women with breast cancer–a population-based study. Acta-Oncologica. 2011;50(7):12. 21604959 10.3109/0284186X.2011.577446

[25] Jassim G, and Whitford D. Quality of life of Bahraini women with breast cancer: a cross sectional study. BMC. 2013;13(212):14. 10.1186/1471-2407-13-212 PMC3644231 23622020

[26] Lôbo S, Fernandes A, Almeida P, Carvalho C, and Sawada N. Quality of life in women with breast cancer undergoing chemotherapy Acta Paul Enferm. 2014;27(6):6.

[27] Dubashi B, Vidhubala E, Xyriac S, and Sagar T. Quality of life among young women with breast cancer: Study from a tertiary cancer institute in south India. Indian Journal of Cancer. 2010;47(2):5. 10.4103/0019-509X.63005 20448376

[28] Nakitare S. Health Related Quality of Life of Breast Cancer Patients at Kenyatta National Hospital. University of Nirobi Masters of medicine Thesis. 2002:78.

[29] Yan B, Yang L, Hao L, Yang C, Quan L, Wang L, et al Determinants of Quality of Life for Breast Cancer Patients in Shanghai,China. PLoS ONE. 2016;11(4):14. 10.1371/journal.pone.0153714 PMC4833339 27082440

[30] Costanzo E, Lutgendorf S, Mattes M, Trehan S, Robinson C, and Tewfik F. Adjusting to life after treatment: distress and quality of life following treatment for breast cancer. Br J Cancer. 2007;97:7. 10.1038/sj.bjc.6604091 PMC2360272 18000503

[31] Mohsin S, UrRehman M, Azam N, and Mashhadi S. comparison of Quality of Life Of Cancer Patients Undergoing Chemotherapy In Tertiary Care Hospital, Rawalpindi. Pak Armaed Forces Med J. 2015;66(1):5.

[32] Foubert J. Cancer-related anaemia and fatigue: assessment and treatment Nursing standard. 2006;20(36):8. 10.7748/ns2006.05.20.36.50.c4154 16755894

[33] Smets E, and Visser M. Fatigue, depression and quality of life in cancer patients: how are they related? Suppport care cancer. 1998;6:8. 10.1007/s005200050142 9540167

[34] Ishak W, Bagot K, Thomas S; Maakian N, Bedwani D, Larson D, et al Quality of Life in Patients Suffering from Insomnia. Innov Clin Neurosci. 2012;9(12):16. 23198273 PMC3508958

