# Supplementary material for: Quality of life and associated factors among patients with breast cancer under chemotherapy at Tikur Anbessa specialized hospital, Addis Ababa, Ethiopia
Source: PLoS One. 2019 Sep 20;14(9):e0222629. doi: 10.1371/journal.pone.0222629 (PMC6754151; doi:10.1371/journal.pone.0222629)
Supplement: S1 File — (DOCX) [file pone.0222629.s001.docx]

## Information sheet (English version)

**Addis Ababa University College of Health Sciences School of Public Health**

**Assessment of breast cancer patient’s quality of life during chemotherapy session at Tikur Anbessa specialized hospital oncology unit Addis Ababa Ethiopia.**

**Introduction**

Greeting, My Name is -------------------; I am working in ---------------------------------. I am working as data collector in a study conducted by the research team member of Addis Ababa University College of Health Sciences School of public Health. I would like to inform you that I would have a short interview concerning this study. Before we go to our discussion, I will ask you to listen carefully to what I am going to tell you about the purpose and general condition of the study and tell me whether you agree or disagree to participate in this study.

The objective of this study is to assess the quality of life of breast cancer patients under chemotherapy treatment session. You are selected to be one of the participants in this study. The study will be conducted through interview.

Apart from the time you are going to use during the interview, there will not be any risk acquired by participating in the study.

Benefits of the study: Taking part in the study helps;

- To improve the knowledge about quality of life of breast cancer patients during chemotherapy treatment session
- To provide basic information for health policy makers, administrators, researchers and for patients who are suffering from breast cancer.

Personal information you are going to give during the data collection will be confidential. Your name will not be written in the questionnaire and once the data is entered into a computer, it will be coded and becomes unidentifiable. Information in the computer will be password protected. Hard copy (paper) documents such as consent and information forms will be kept in a secured locked cabinet

You will be recruited based on your willingness and without obligation to participate in the study. You have the right to withdraw from participating in the study whenever you want to (before completing the study). Participation in the study will have no implications for your relation and treatment at the hospital.

Are you willing to participate in the study? 1. Yes 2. No

Thank you!

## Informed consent form (English version)

I understand all the information provided to me by the data collector, and I am willing to participate in the interview.

Signature ------------------------- Date ------------------

## English version of the Questionnaire

| 1. Socio demographic data | | | |
| --- | --- | --- | --- |
|  | Variables | Response | |
| 1.1 | Age | ------- years | |
| 1.2 | Educational status | 1. Illiterate 2. Read and write 3. Primary education 4. Secondary education 5. College and above | |
| 1.3 | occupation | 1. Housewife 2. Governmental 3. Non-governmental 4. Private | |
| 1.4 | Religion | 1. Orthodox Christian 2. Muslim 3. Protestant 4. Catholic 5. Other ----- | |
| 1.5 | Residence | 1. Addis Ababa 2. Out of Addis Ababa | |
| 1.6 | Marital status | 1. Married 2. Single 3. Divorced 4. Widowed | |
| 1.7 | Monthly income | ------------ | |
| 1. **Clinical data** | | | |
| 2.1 | How many cycles of chemotherapy treatment have you taken | | 1. Two 2. three 3. four 4. five 5. six 6. seven 7. eight |
| 2.2 | Have you taken cancer treatment before? | | 1. Yes 2. No |
| 2.3 | If yes, for question number 2.2, what type of treatment have you taken? | | 1. Surgery 2. Radiotherapy 3. Chemotherapy 4. Hormonal therapy |
| 2.4 | Stage of tumor | | 1. Stage I 2. Stage II A 3. Stage IIB 4. Stage IIIA (T3,N1,M0) 5. Stage III * 6. Stage IV |
| 2.5 | Tumor size | | 1. Tx (Not assessed) 2. T1 (< 2 cm) 3. T2 (2 cm-5 cm) 4. T3 (> 5 cm) 5. T4 (Any size with extension to chest wall) |
|  | Nodal involvement | | 1. N0 2. N1 3. N2 4. N3 5. N4 |
| 2.6 | Do you have Comorbid diseases | | 1. Yes 2. No |
|  | If yes for question number 2.6, what type of disease | | 1. Hypertension 2. Diabetes mellitus 3. Heart disease 4. Other--------- |

1. **EORTC QLQ C-30 (English version)**

We are interested in some things about you and your health. Please answer all of the questions yourself by circling the number that best applies to you. There are no "right" or "wrong" answers. The information that you provide will remain strictly confidential.

Please fill in your initials …………….

Code number ………………..

Date ……………………..

1. Do you have any trouble doing strenuous activities, like carrying a heavy shopping bag or a suitcase?

1. Not at all 2. A little 3. Quite a bit 4. Very much

1. Do you have any trouble taking a long walk?

1. Not at all 2. A little 3. Quite a bit 4. Very much

1. Do you have any trouble taking a short walk outside of the house?

1. Not at all 2. A little 3. Quite a bit 4. Very much

1. Do you need to stay in bed or a chair during the day?

1. Not at all 2. A little 3. Quite a bit 4. Very much

1. Do you need help with eating, dressing, washing yourself or using the toilet?

1. Not at all 2. A little 3. Quite a bit 4. Very much

**During the past week**

1. Were you limited in doing either your work or other daily activities?

1. Not at all 2. A little 3. Quite a bit 4. Very much

1. Were you limited in pursuing your hobbies or other leisure time activities?

1. Not at all 2. A little 3. Quite a bit 4. Very much

1. Were you short of breath?

1. Not at all 2. A little 3. Quite a bit 4. Very much

1. Have you had pain?

1. Not at all 2. A little 3. Quite a bit 4. Very much

1. Did you need to rest?

1. Not at all 2. A little 3. Quite a bit 4. Very much

1. Have you had trouble sleeping?

1. Not at all 2. A little 3. Quite a bit 4. Very much

1. Have you felt weak?

1. Not at all 2. A little 3. Quite a bit 4. Very much

1. Have you lacked appetite?

1. Not at all 2. A little 3. Quite a bit 4. Very much

1. Have you felt nauseated?

1. Not at all 2. A little 3. Quite a bit 4. Very much

1. Have you vomited?

1. Not at all 2. A little 3. Quite a bit 4. Very much

1. Have you been constipated?

1. Not at all 2. A little 3. Quite a bit 4. Very much

1. Have you had diarrhea?

1. Not at all 2. A little 3. Quite a bit 4. Very much

1. Were you tired?

1. Not at all 2. A little 3. Quite a bit 4. Very much

1. Did pain interfere with your daily activities?

1. Not at all 2. A little 3. Quite a bit 4. Very much

1. Have you had difficulty in concentrating on things, like reading a newspaper or watching television?

1. Not at all 2. A little 3. Quite a bit 4. Very much

1. Did you feel tense?

1. Not at all 2. A little 3. Quite a bit 4. Very much

1. Did you worry?

1. Not at all 2. A little 3. Quite a bit 4. Very much

1. Did you feel irritable?

1. Not at all 2. A little 3. Quite a bit 4. Very much

1. Did you feel depressed?

1. Not at all 2. A little 3. Quite a bit 4. Very much

1. Have you had difficulty remembering things?

1. Not at all 2. A little 3. Quite a bit 4. Very much

1. Has your physical condition or medical treatment interfered with your family life?

1. Not at all 2. A little 3. Quite a bit 4. Very much

1. Has your physical condition or medical treatment interfered with your social activities?

1. Not at all 2. A little 3. Quite a bit 4. Very much

1. Has your physical condition or medical treatment caused you financial difficulties?

1. Not at all 2. A little 3. Quite a bit 4. Very much

**For the following questions please circle the number between 1 and 7 that best applies to you**

1. How would you rate your overall health during the past week?

1 2 3 4 5 6 7 **very poor excellent**

1. How would you rate your overall quality of life during the past week?

1 2 3 4 5 6 7 **very poor excellent**

1. **EORTC QLQ -BR23 (English version)**

Patients sometimes report that they have the following symptoms or problems. Please indicate the extent to which you have experienced these symptoms or problems during the past week.

**During the past week**

1. Did you have dry mouth

1. Not at all 2. A little 3. Quite a bit 4. Very much

1. Did food and drink taste different than usual?

1. Not at all 2. A little 3. Quite a bit 4. Very much

1. Were your eyes painful, irritated or watery?

1. Not at all 2. A little 3. Quite a bit 4. Very much

1. Have you lost any hair?

1. Not at all 2. A little 3. Quite a bit 4. Very much

1. Answer this question only if you had any hair loss. were you upset by the loss of your hair?

1. Not at all 2. A little 3. Quite a bit 4. Very much

1. Did you feel ill or unwell?

1. Not at all 2. A little 3. Quite a bit 4. Very much

1. Did you have hot flushes?

1. Not at all 2. A little 3. Quite a bit 4. Very much

1. Did you have headache?

1. Not at all 2. A little 3. Quite a bit 4. Very much

1. Have you felt physically less attractive as a result of your disease or treatment?

1. Not at all 2. A little 3. Quite a bit 4. Very much

1. Have you been feeling less feminine a result of your disease or treatment?

1. Not at all 2. A little 3. Quite a bit 4. Very much

1. Did you find it difficult to look at yourself naked?

1. Not at all 2. A little 3. Quite a bit 4. Very much

1. Have you been dissatisfied with your body?

1. Not at all 2. A little 3. Quite a bit 4. Very much

1. Were you worried about your health in the future?

1. Not at all 2. A little 3. Quite a bit 4. Very much

**During the past four weeks**

1. To what extent were you interested in sex?

1. Not at all 2. A little 3. Quite a bit 4. Very much

1. To what extent were you sexually active? (with or without intercourse)

1. Not at all 2. A little 3. Quite a bit 4. Very much

1. Answer this question only if you have been sexually active. To what extent was sex enjoyable for you?

1. Not at all 2. A little 3. Quite a bit 4. Very much

1. Did you have any pain in your arm or shoulder?

1. Not at all 2. A little 3. Quite a bit 4. Very much

1. Did you have a swollen arm or shoulder?

1. Not at all 2. A little 3. Quite a bit 4. Very much

1. Was it difficult to raise your arm or to move it sideways?

1. Not at all 2. A little 3. Quite a bit 4. Very much

1. Have you had any pain in the area of your affected breast?

1. Not at all 2. A little 3. Quite a bit 4. Very much

1. Was the area of your affected breast swollen?

1. Not at all 2. A little 3. Quite a bit 4. Very much

1. Was the area of your affected breast oversensitive?

1. Not at all 2. A little 3. Quite a bit 4. Very much

1. Have you had skin problems on or in the area of your affected breast (e.g, itchy, dry, flaky)?

1. Not at all 2. A little 3. Quite a bit 4. Very much

## Information sheet (Amharic version)

**አድሲ አበባ ዩኒቨርሲቲ ህክመናና ጤና ሳይንስ ኮሌጅ የማህበረሰብ ትምህርት ክፍል**

**የተሳታፊዎች መረጃ ቅፅ**

ጤና ይስጥልኝ፣ ስሜ…………….ይባላል. የምስራዉ…………………..ነዉ. በአዲስ አበባ ዩኒቨርሲቲ ህክምናና ጤና ሳይንስ ኮሌጅ የጥናት ቡድን አባላት በተዘጋጀዉ ጥናት ላይ መረጃ ሰብሳቢ ሁኜ እየሰራሁ ነዉ.ይህን ጥናት በተመለከተ አጭር ቃለመጠይቅ ከርስዎ ጋር ይኑረኛል፡፡ ከቃለ ምልልሱ በፊት ግን ስለዚህ ጥናት አላማ በአጭሩ እገልጽሎታለው፡፡ ይህ ጥናት በአ.አ.ዩ ህክምናና ጤና ሳይንስ ኮሌጅ የ ማስተርስ ፕሮግራም መመረቂያ ፅሁፍ ነዉ፡፡ የዚህ ጥናት አላማ በጥቁር አንበሳ ስፔሻላይዝድ ሆስፒታል የጡት ካንሰር ላለባቸውና በኬሞ ቴራፒ ህክምና ላይ ላሎ ህመምተኞች በህይወት የመኖር ጣዕም ላይ የሚያተኩር ነው፡፡ እርሶም በዚህ ጥናት ላይ እንዲሳተፉ ተጋብዘዋል፡፡አሁን የመናደርገው ቃለ ምልልስ ምክንያት ጊዜዎን ከመሻማት ውጪ በአካሉ ላይ የሚደረግ ምንም አይነት የለም ፡፡

**የዚህ ጥናት ጥቅም**

• የጡት ካንሰር ያለባቸውና ኬሞ ቴራፒ ህክምና ላይ ላሎ ህመምተኞች በህይወት የመኖር ጣዕም እዉቀት ይጨምራል፡፡

• የዚህ ጥናት ውጤት ሌሎች ጤናን በተመለከተ መተዳደሪያ ደንብ ለሚያወጡ ግለሰቦች አስተዳደሮች ጥናትን ለሚያከናውኑ ግለሰቦች በጡት ካንሰር ለሚሰቃዩ ህሙማን መረጃ ይሰጣል፡፡

ይህ የሚሰጡን ግላዊ መረጃ ሚስጥራዊነቱ የተጠበቀ ነው ፡፡ ይህ መረጃ በኮምፒውተር በሚስጥር ከተመዘገበ በኃላ ስምዎት አይጠቀስም እናም በምንም አይት ምነገድ ሊታወቅ አይችልም፡፡ በኮምፒውተር ውስጥ ያለው መረጃ በሚስጥር ኮድ ታስሮ ይቀመጣል፡፡

ይህን መረጃ የሚሰጡን ያለምንም ግዴታ በሙሉ ፍቃደኝነት ነው ከተጀመረ በኃላ በማንኛውም ግዜ ተሳትፎሁን የማቁረጥ ሙሉ መብት ይኖሮታል፡፡ እርሶዎ በጥናቱ በመሳተፎ ከሆስፒታል ካልዎት ግንኞነት ጋር ተፅእኖ የለውም

በጥናቱ ላይ ለመሳተፍ ፍቃደኛ ነዎት?

1. አዎ 2. አይደለውም

አመሰግናለው!!

- 1. Informed consent form (Amharic version)

**ፈቃደኝነትን የሚያረጋግጥ ቅፅ**

በመረጃ ሰብሳቢዋ መሰረት የተነገረኝን መረጃ በሙሉ ተረድቻለሁ፡፡ አናም በዚህ ቃለመጠይቅ ላይ ለመሳተፍ ፈቃደኛ ነኝ፡፡

የምላሽ ሰጪ ፊርማ .............................................

ቀን……………………………….

የጠያቂ ፊርማ …………………………………….

## 7.8. **Amharic version of questionnaire**

1. **ማንነትን የሚመለከት ጥያቄዎች**

1.1 ዕድሜ ………………………

1.2 የትምህርት ደረጃ ………….ሀ/ አልተማርኩም

ለ/ ማንብብ መፃፍ እችላላሁ

ሐ/አንደኛ ደረጃ ያጠናከኩ

መ/ ሁለተኛ ደረጃ ያጠናከኩ

ሠ/ ኮሌጅ እና ከዚያ በላይ

ረ/ሌላ ካለ

1.3 ስራ ሀ/ የቤት እመቤት

ለ/ የመንግስት ሠራተኛ

ሐ/ መንግስታዊ ያልሆነ ድርጅት ሠራተኛ

መ/ ግል

1.4 ሀይማኖት…………………ሀ/ ኦርቶዶክስ ክርስቲያን

ለ/ሙስሊም

ሐ/ፖሮቴስታንት

መ/ካቶሊክ

ሠ/ ሌላ ካለ

1.5 የመኖሪያ ቦታ………………ሀ/ አድስ አበባ

ለ/ **ከ**አድስ አበባ ውጭ

1.6 የጋብቻ ሁኔታ …………….ሀ/በትዳር ላይ ያሉ

ለ/ ያላገባች

ሐ/የፈታች

መ/ ባል የምተባት

1.7 የወር ገቢ…………………………

**2. የሕክምና መረጃ/ ከበሽተኛ ካርድ የሚወሰድ**

2.1. ለስንተኛ ዙር ነው ይህን መድሀኒት የሚወስዱት

ሀ/ለሁለተኛ ጊዜ መ/ ለአምተኘ ጊዜ

ለ/ለሶስተኛ ጊዜ ሠ/ለስድስተኛ ጊዜ

ሐ/ ለአራተኛ ጊዜ ረ/ ለሰባተኛ ጊዜ

ሰ/ ለስምተኛ ጊዜ

2.2. ከዚህ ህክምና በፊት ሌላ የካንሰር ህክምና ወስደዋል?

ሀ/ አዎ ወስደዋል

ለ/ አልወስዱም

2.3. ለ2 .2 መልስም አዎ ከሆነ ምን አይነተ ህክምና ነው የወሰዱት ?

ሀ/ ቀዶ ህክምና

ለ/የጨረር ህክምና

2.4. የዕጢው ደረጃ ሀ/ Stage I

ለ/ Stage II A

ሐ/ Stage IIB

መ/ Stage IIIA (T3,N1,M0)

ሠ/ Stage III

ረ/ Stage IV

የዕጢዉ መጠን ሀ/ Tx (Not assessed)

ለ/ T1 (< 2 cm)

ሐ/ T2 (2 cm-5 cm)

መ/ T3 (> 5 cm)

ሠ/ T4 (Any size with extension to chest wall)

- 1. ተጉዋዳኝ በሺታ ዐለብዎት ሀ/ አዎ

ለ/ የለም

- 1. ምን አይነተ ሀ/ የደም ግፊት

ለ/ የስኳረ በሺታ

ሐ/የልብ በሺታ

መ/ሌላ

1. **EORTC QLQ C-30 (Amharic version)**

የእኛው ቡድን ስለከእርሶዎ ስለጤንነትዎ ልዩ ትኩረት መስጠት ነው፡፡

እባክዎትን የሚከተሉትን ጥያቄዎች በሙሉ እርሶዎ ትክከለኛ ብለው ያመኑበትን በማክበብ ይመልሱ ፡፡ ትክክለኛ መልስ ወይም የተሳሰተ መልስ የሚባል የለም፡፡ የሚሰጡት መረጃ ሁሉ ሚስጠራዊነቱ በደንብ የተጠበቀ ይሆናል፡፡

እባካዎን የእርሶዎንና የአያትዎን የስም መጀመሪያ ፊደል ይንገሩኝ…………

መለያቁጥር…………………

ቀን፡………

|  |  | በጭራሽ | በትንሹ | በመጠኑ | በብዛት |
| --- | --- | --- | --- | --- | --- |
| 3.1 | ከባድ ስራ ወይም እንቅስቃሴ ለመስራት ችግር አለብዎ (ለምሳሌ፤ዘንቢል ለመሽከም) | 1 | 2 | 3 | 4 |
| 3.2 | ረጅም የእግር ጉዞ ለማድረግ ችግር አለብዎ | 1 | 2 | 3 | 4 |
| 3.3 | አጭር የእግር ጉዞ ለማድረግ ችግር አለብዎ(ከቤትዎ ውጪ) | 1 | 2 | 3 | 4 |
| 3.4 | በህመምዎ የተነሳ በቀን አልጋ ላይ ወይም ወንበር ላይ ሁነው ረዘም ላለ ሰዓት ያሳልፍሉ? | 1 | 2 | 3 | 4 |
| 3.5 | የዕለት ተዕለት እንቅስቃሴዎን ለማከናውን ረዳት ወይም አጋዥ ይፈልጋሉ ?ለምሳሌ መመገብ መልበስ | 1 | 2 | 3 | 4 |
| 3.6 | ስራዎትን ወይም የዕለት ተዕለት እንቅስቃሴዎን ለማከናወወን አግድዎት ነበር | 1 | 2 | 3 | 4 |
| 3.7 | በትርፍ ጊዜ የሚከናወን ስራ ወይንም ዝነንባሌዎን ለማሳካት ወይም ሌሎች የመዝናኛ ጊዜዎች ለማሳለፍ አግዶዎታለ? | 1 | 2 | 3 | 4 |
| 3.8 | ሲተነፍሱ ትንፋሽ ማጠር አጋትጥመዎት ነበር? | 1 | 2 | 3 | 4 |
| 3.9 | የህመም ስሜት ነበረብዎ? | 1 | 2 | 3 | 4 |
| 3.10 | ከወትሮዎ የተለየ ዕረፍት አስፈልጎዎት ነበር? | 1 | 2 | 3 | 4 |
| 3.11 | የእንቅልፍ ችግር ነበረብዎ? | 1 | 2 | 3 | 4 |
| 3.12 | አቅም ያንስዎት ነበር? | 1 | 2 | 3 | 4 |
| 3.13 | የምግብ ፍላጎትዎ ቀንሷል? | 1 | 2 | 3 | 4 |
| 3.14 | የማቅለሽለሽ ስሜት ነበረበዎ? | 1 | 2 | 3 | 4 |
| 3.15 | አስመልስዎት ነበር ? | 1 | 2 | 3 | 4 |
| 3.16 | የሰገራ ድርቀት ነበረብዎ ? | 1 | 2 | 3 | 4 |
| 3.17 | ተቅማጥ ነበረብዎ? | 1 | 2 | 3 | 4 |
| 3.18 | የድካም ስሜት ነበረብዎት ? | 1 | 2 | 3 | 4 |
| 3.19 | ህመሙ ዕለት ተዕለት እንቅስቃሴዎን  ያውክብዎ ነበረ ? | 1 | 2 | 3 | 4 |
| 3.20 | አንዳንድ ነገሮች ትኩረት ስጥተው ለመስራት? ያዉክዎት ነበር? (ለምሳሌ፤ ጋዜጣ ለማንበብ፤ ራዲዩ በማዳመጥ ) | 1 | 2 | 3 | 4 |
| 3.21 | የውጥረት ስሜት ነበረብዎ ? | 1 | 2 | 3 | 4 |
| 3.22 | የመጨነቅ ስሜት ነበረብዎ ? | 1 | 2 | 3 | 4 |
| 3.23 | የመነጫነጭ ስሜት ነበረብዎ? | 1 | 2 | 3 | 4 |
| 3.24 | የመደበር ስሜት ነበረብዎ ? | 1 | 2 | 3 | 4 |

**ባለፈው ሳምንት ውስጥ**

|  | |  | | በጭራሽ | | | በትንሹ | | | በመጠኑ | | | በብዛት |
| --- | --- | --- | --- | --- | --- | --- | --- | --- | --- | --- | --- | --- | --- |
| 3.25 | | ነገሮችን የማስታወስ ችግር ነበረብዎ ? | | 1 | | | 2 | | | 3 | | | 4 |
| 3.26 | | የጤናዎ ሁኔታ ወይም የሚከታተሉት  ህክምና ኑሮ ላይ ያሳደረው ተፅዕኖ አለ | | 1 | | | 2 | | | 3 | | | 4 |
| 3.27 | | የጤናዎ ሁኔታ ወይም የሚከታተሉት ህክምና በማህበራዊ ህይወትዎ፤ በሚያደርጉት እንቅስቃሴ ላይ ያሳደረው ተፅእኖ አለው ? | | 1 | | | 2 | | | 3 | | | 4 |
| 3.28 | | የጤናዎ ሁኔታ ወይም የሚከታተሉት ህክምና ገንዘብ እንዲያጥርዎ /እንደቸገርዎ አድርጓል ? | | 1 | | | 2 | | | 3 | | | 4 |
|  |  | | በጣም መጥፎ | |  |  | |  |  | |  | እጅግ በጣም ጥሩ | |
| 3.29 | ባለፈዉ ሳምንት በአጠቃላይ የጤንነት ሁኔታዎን እንዴት ይመዝኑታል? | | 1 | | 2 | 3 | | 4 | 5 | | 6 | 7 | |
| 3.30 | ባለፈዉ ሳምንት የርስዎን የሂወት የመኖር ጣዕም ዕንዴት ይመዝኑታል? | | 1 | | 2 | 3 | | 4 | 5 | | 6 | 7 | |

1. **EORTC QLQ BR-23 (Amharic version)**

|  |  | በጭራሽ | | በትንሹ | | | በመጠኑ | | በብዛት |
| --- | --- | --- | --- | --- | --- | --- | --- | --- | --- |
| 4.1 | የአፍ ድርቀት ነበረብዎ ? | 1 | | 2 | | | 3 | | 4 |
| 4.2 | ምግብና መጠጥ ከወትሮ የተለያ ጣዕም ተለዉቶብዎ ነበር? | 1 | | 2 | | | 3 | | 4 |
| 4.3 | አይንዎን የህምም የመቆርቆር ወይም ውሀ የማዘል ስሜት ነበረብዎ ? | 1 | | 2 | | | 3 | | 4 |
| 4.4 | ፀጉርዎ ሳስቶ ፤ ተነቅሉ ነበር? | 1 | | 2 | | | 3 | | 4 |
| 4.5 | ለጥያቄ ቁጥር 4.4 መልስዎአዎ ከሆነ ወይም ከ ምርጫ ቁጥር 1 ውጪ ከሆነ ፀጉራ በመነቀሉ ተናደው ነበር? | 1 | | 2 | | | 3 | | 4 |
| 4.6 | የህምም ስሜት ነበረብዎ? | 1 | | 2 | | | 3 | | 4 |
| 4.7 | ፊትዎ አካባቢ የሙቀት፤ የማቃተል ስሜት ነበርብዎ? | 1 | | 2 | | | 3 | | 4 |
| 4.8 | ራስ ምታት ነበረብዎ ? | 1 | | 2 | | | 3 | | 4 |
| 4.9 | በጤናዎ ሁኔታ ወይም በሚከታተሉት ህክምና ምክንያት አይን የማይስቡ ሰዉ እንደሆኑ ተሰምቶዎት ነበር ? | 1 | | 2 | | | 3 | | 4 |
| 4.10 | በጤናዎ ሁኔታ ወይም በሚከታተሉት ህክምና ምክንያት ሴትነትዎ ተሰምቶዎት ነበር? | 1 | | 2 | | | 3 | | 4 |
| 4.11 | እርቃንዎን ሁነው ራስዎን መመልከት ከብደዎት ነበር? | 1 | | 2 | | | 3 | | 4 |
| 4.12 | በአካልዎ ወይም በሰውነትዎ አቋም ያለመርካትና ያለመደሰት ስሜት ተሠምቶዎት ነበር? | 1 | | 2 | | | 3 | | 4 |
| 4.13 | ስለወደፊት ጤንነትዎ ተጨንቀው ነበር? | 1 | | 2 | | | 3 | | 4 |
|  |  |  | |  | | |  | |  |
|  |  |  | |  | | |  | |  |
|  |  |  | |  | | |  | |  |
| ባለፈት አራት ሳምንት ውስጥ | | | | | | | | | |
|  | | | | | | | | | |
|  |  | | በጭራሽ | | በትንሹ | በመጠኑ | | በብዛት | |
| 4.14 | ለፆታዊ ግንኙንት ምን ያህል ፍላጉት ነበርዎት ? | | 1 | | 2 | 3 | | 4 | |
| 4.15 | ፆታዊ ግንኙነት ላይ ምን ያህል ተሳታፊ ነበሩ? (በግብረ ስጋ ግንኙነት ወይም ከግንኙነት ውጭ)? | | 1 | | 2 | 3 | | 4 | |
| 4.16 | ለጥያቄ ቁጥር 4፣15 መልስዎ ከ 1 ወጪ ከሆነ (ግብረ ስጋ ግንኙነቱ ምን ያህል አስደሳች ነበር? | | 1 | | 2 | 3 | | 4 | |
| 4.17 | ክንድዎ ላይ ወይም ትከሻዎ ላይ ህመም ተስምቶዎት ነበር ? | | 1 | | 2 | 3 | | 4 | |
| 4.18 | የክንድ ወይም ትከሻ እብጠት ነበረብዎት? | | 1 | | 2 | 3 | | 4 | |
| 4.19 | ክንድዎን ለማነሳት ወይም ወደ ጉን ለማንቀሳቀስ ተቸግረው ነበር? | | 1 | | 2 | 3 | | 4 | |
| 4.20 | በበሽታ የተጠቃዉ ጡትዎ አካባቢ የህምም ስሜት ነበረብዎ? | | 1 | | 2 | 3 | | 4 | |
| 4.21 | በበሽታ የተጠቃዉ ጡትዎ አካባቢ እብጠት ነበረብዎ ? | | 1 | | 2 | 3 | | 4 | |
| 4.22 | በበሽታ የተጠቃዉ ጡትዎ አካባቢ በትንሹ ሲነካ ከባድ የህመም ስሜት ነበረብዎ? | | 1 | | 2 | 3 | | 4 | |
| 4.23 | በበሽታ የተጠቃዉ ጡትዎ አካባቢ የቆዳ ችግር ነበረብዎ? | | 1 | | 2 | 3 | | 4 | |

**Curriculum Vite**

1. **Personal data**

1.1 Name Anissa Mohammed Hassen

1.2 Permanent Address Addis Ababa

1.3 E-Mail Address anisa.moh26@gmail.com

1.4 Gender Female

1.5 Nationality Ethiopian

1.6 Date of Birth November 24, 1986 E.C

1.7 Place of Birth South Wello, Kombolcha

1.8 Tel. +251921257471

1. **Educational background**
   1. From grade 1-8 Kombolcha Junier Primary School.
   2. From grade 9_10 Kombolcha Millennium Secondary school.
   3. From grade 11-12 Kombolcha secondary and preparatory School.
   4. Higher Education in university of Gondar Health Science College .
2. **Qualifications**

- BCS Degree in Public Health Officer from University of Gondar in 2007 EC.

1. **Work experience**

* worked for a year as a graduate assistance in Wello university.

1. **Language skill**

- Excellent Speaking ,Reading , Writing and Listing In Amharic and good in English

1. **Other skills**

- Basic ADULT And Pediatric ART Care Training

1. **Reference**

- Available Up on request

| **ASSURANCE OF PRINCIPAL INVESTIGATOR**  The undersigned agrees to accept responsibility for the scientific ethical and technical  Conduct of the research project and for provision of required progress reports as  Per terms and conditions of the Research Publications Office in effect at the time of  Grant is forwarded as the result of this application.    Name of the student: _______________________________________  Date.____________________ Signature _________________ |
| --- |
| **Approval of the primary Advisor**    Name of the primary advisor:_________________________________  Date.____________________ Signature _________________ |
